# RNA methylation in vascular disease: a systematic review

**DOI:** 10.1186/s13019-022-02077-1

**Published:** 2022-12-19

**Authors:** Yue Shu, Yilong Guo, Yin Zheng, Shuwu He, Zhensu Shi

**Affiliations:** 1 Geriatric Multi-Clinic Center, Hainan ChengMei Hospital, Haikou, Hainan People’s Republic of China; 2 Department of Special Medical Services, Hainan Cancer Hospital, Haikou, Hainan People’s Republic of China; 3 Medical School of Chinese PLA, Beijing, People’s Republic of China; 4 Department of Vascular and Endovascular Surgery, The First Medical Centre of Chinese PLA General Hospital, Beijing, People’s Republic of China; 5 Department of Cardiovascular Surgery, The Second Affiliated Hospital of Hainan Medical University, 48th of Bai Shui Tang Road, Haikou, 570311 Hainan People’s Republic of China

**Keywords:** N6-methyladenosine, RNA-modifying enzymes, Risk factors of vascular disease, Vascular disease

## Abstract

Despite the rise in morbidity and mortality associated with vascular diseases, the underlying pathophysiological molecular mechanisms are still unclear. RNA N6-methyladenosine modification, as the most common cellular mechanism of RNA regulation, participates in a variety of biological functions and plays an important role in epigenetics. A large amount of evidence shows that RNA N6-methyladenosine modifications play a key role in the morbidity caused by vascular diseases. Further research on the relationship between RNA N6-methyladenosine modifications and vascular diseases is necessary to understand disease mechanisms at the gene level and to provide new tools for diagnosis and treatment. In this study, we summarize the currently available data on RNA N6-methyladenosine modifications in vascular diseases, addressing four aspects: the cellular regulatory system of N6-methyladenosine methylation, N6-methyladenosine modifications in risk factors for vascular disease, N6-methyladenosine modifications in vascular diseases, and techniques for the detection of N6-methyladenosine-methylated RNA.

## Background

RNA modification is a ubiquitous process in nature that regulates the transmission and expression of genetic information in cells. It can affect the phenotype of cells by influencing the transcription, splicing, stability, transnuclear transport, and translation of RNA [1–3]. According to the MODOMICS [4] database, more than 170 chemical modifications that regulate RNA have been discovered to date, most involving methylation. The methylation of RNA results in various forms, including: N1-methyladenosine (m1A), 5-methylcytosine (m5C), N6-methyladenosine (m6A), 7-methylguanosine (m7G), N1-methyladenosine (m6Am), and 2ʹ-O-methylation (2ʹ-OMe) [4]. Moreover, RNA methylation modifications can be found in several different types of RNA, such as transfer (tRNA), ribosomal (rRNA), messenger (mRNA), and non-coding (ncRNA) RNAs, and regulate their expression [5, 6].

M6A methylation, the addition of a methyl group at the nitrogen atom at position 6 of adenine, is catalysed by methyltransferases and is the most common modification of mRNA in eukaryotic cells [7–9]. Previous studies have shown that RNA m6A modification plays an important role in the development, progression, and prognosis of cardiovascular diseases [10–13]. However, reports focused on cardiac disease. In recent years, with economic development, the morbidity due to vascular diseases, especially critical diseases such as aortic dissection (AD) and aortic aneurysm, has been increasing, which endangers human life and health. To better prevent and treat vascular diseases, intensive research on their underlying mechanisms is critical [14–16]. However, studies that summarize the role of RNA methylation in the progression of vascular diseases are still lacking. Therefore, in this review, we discuss the role of RNA m6A modifications in risk factors, morbidity, and progression of vascular diseases, and provide a direction for the research of m6A modifications in the field of vascular diseases.

## Main text

### The RNA m6A modification system

Previous studies have found that m6A is generally present in specific RNA motifs, such as DRACH (D = A/G/U; R = A/G; H = A/U/C), 3ʹ untranslated regions (UTRs), or around the stop codon [17, 18]. In eukaryotes, RNA N6-methyladenosine exists in a dynamic equilibrium that is determined by a regulatory system consisting of writer, eraser, and reader proteins [19–21]. Writing and erasing of m6A-methylated RNA occur mainly in the nucleus, whereas reading occurs mainly in the cytoplasm [17, 18, 20, 22, 23]. Abnormalities in any component of this regulatory system can lead to disturbances in the balance of RNA m6A modifications, which are associated with a number of diseases, including failure to thrive, obesity, type 2 diabetes mellitus (T2DM), cancer, and weakness [2, 7, 24–29]. The associations between individual components of the m6A modification system and vascular diseases are presented in Table 1.

**Table 1:** Association between individual components of the m6A modification system and vascular diseases

Component		m6A levels	Main function	Reference
Writer	METTL3	Increased	Major catalytic subunit	[32, 33]
	METTL14	Increased	RNA adaptor that enhances METTL3 function	[32, 33]
	METTL16	Increased	Catalytic subunit (on adenosine in loops or secondary structures outside DRACH motif) Regulates the expression of S-adenosyl methionine synthase	[42–44]
	WTAP	Increased	Guides binding of METTL3/METTL14 heterodimer to target RNA Increases DSP and leads to brain arteriovenous malformation lesions	[36–41]
	VIRMA (KIAA1429)	Increased	Catalytic subunit	[45]
Eraser	FTO	Reduced	Role in tumorigenesis, oocyte maturation and adipose tissue regulation	[46–55]
	ALKBH5	Reduced	Fundamental and widespread role within mammalian cells	[46–55]
Reader	YTHDF1	–	Facilitates target mRNA translation (combined with eIF3)	[29]
	YTHDF2	–	Reduces the stability of m6A-methylated mRNA	[2]
	YTHDF3	–	Role in the initiation of translation (through interaction with YTHDF1 and YTHDF2)	[60]
	YTHDC2	–	Facilitates mRNA translation Affects mRNA stability	[61, 62]
	YTHDC1	–	Regulates pre-mRNA splicing and maturation Role in the nucleus transport of methylated mRNAs	[26]
	HNRNPs	–	Alters the structure of target RNA for recognition by RNA binding proteins	[31, 63, 64]

#### Writers of N6-methyladenosine

Writers deposit N6-methyladenosine on target RNAs. The writer complex includes the enzymes methyltransferase-like 3 (METTL3), methyltransferase-like 14 (METTL14), Wilms tumour 1-associated protein (WTAP), methyltransferase-like 16 (METTL16), and vir-like m6A methyltransferase associated (VIRMA). Two proteins, RNA-binding motif protein 15 (RBM15) and RNA-binding motif protein 15B (RBM15B), modulate the action of this complex [30, 31].

Previous studies have shown that METTL3 is the major catalytic subunit of the RNA methyltransferase complex. METTL3 and METTL14 form a heterodimer, in which METTL14 acts as an adaptor protein that enhances the function of METTL3 [32, 33]. Studies by Yao et al*.*[34] and Chamorro-Jorganes et al.[35] have shown that METTL3 is essential for the regulation of the VEGFA and Wnt signalling pathways, which participate in physiological processes such as angiogenesis, vascular permeability, and spermatogonial stem cell maintenance.

WTAP acts as a guide to direct binding of the METTL3/METTL14 heterodimer to the target RNA [36, 37]. Previous studies have shown that desmoplakin (DSP) is a special protein that plays an important role in the formation of the lumen of blood vessels by endothelial cells (ECs). WTAP increases the stability of DSP mRNA through m6A modification; m6A-modified DSP is recognized by and binds insulin-like growth factor 2 mRNA binding proteins 1 (IGF2BP1) and 3 (IGF2BP3), causing a decrease in the morbidity of brain arteriovenous malformation lesions [38–41].

Recent studies have shown that METTL16 is catalytically active in specific situations [42, 43]. Study of Mendel et al*.* have suggested that METTL16 regulates the expression of S-adenosyl methionine synthase, which in turn plays a role in early embryonic development [44].

VIRMA is another recently discovered catalytic component of the RNA methyltransferase complex that deposits N6-methyladenosine in the 3ʹUTR [45].

#### Erasers of m6A

Erasers are demethylates that eliminate RNA m6A modifications. So far, two important erasers have been identified: the fat-mass and obesity-associated protein (FTO) and α-ketoglutarate-dependent dioxygenase AlkB homolog 5 (ALKBH5). Both are members of the α-ketoglutarate-dependent dioxygenase family of enzymes; however, while FTO is expressed in all tissues, ALKBH5 is primarily expressed in the testes [46–48] and their targets also differ: FTO is located between the nucleus and cytoplasm and has a preference for m6Am, m6A, and m1A; ALKBH5 is located in the nucleus and has a preference for m6A [49, 50]. The FTO and ALKBH5 enzymatic activity is dependent on Fe^2 +^ and α-ketoglutarate as cofactors [51, 52]. FTO plays a role in tumorigenesis, oocyte maturation, and adipose tissue regulation, whereas ALKBH5 plays a fundamental and widespread role within mammalian cells [7, 52–55].

#### Readers of m6A

Readers are proteins that can identify, bind, and link RNA N6-methyladenosine modifications to specific biological functions. Many readers have been recognized, including the YTH family (YTHDF1, YTHDF2, YTHDF3, YTHDC1, and YTHDC2) [56], the heterogeneous nuclear ribonucleoprotein family (HNRNPA2/B1, HNRNPC, and HNRNPG) [57], eukaryotic initiation factor 3 (eIF-3), insulin-like growth factor 2 mRNA binding proteins (IGF2BPs 1, 2, and 3) and proline rich coil 2A protein (Prr2a). They can directly bind m6A or act as part of m6A-binding ribonucleoprotein complexes to exert their biological effects [58, 59].

YTHDF1, YTHDF2, YTHDF3, and YTHDC2 are localized mainly in the cytoplasm. Previous studies have shown that YTHDF1 combines with eIF-3 to facilitate target mRNA translation [29]. YTHDF2 reduces the stability of m6A-modified mRNA [2], and YTHDF3 plays an important role at the initiation of translation through interaction with both YTHDF1 and YTHDF2 [60]. YTHDC2 facilitates mRNA translation and affects its stability [61, 62]. YTHDC1 is located in the nucleus, regulates pre-mRNA splicing and maturation, and plays a key role in the nucleus transport of methylated mRNAs [26].

Members of the HNRNP family process RNAs through the “m6A switch” system, where the structure of the target RNA is changed and recognized by RNA binding proteins [31, 63, 64].

### RNA m6A modifications in risk factors for vascular disease

In recent years, the morbidity of vascular diseases has gradually increased; common high-risk factors of vascular diseases, including obesity, diabetes, hypertension, and atherosclerosis, have played a critical role in this rise. These factors can cause coronary heart disease (CHD), arterial occlusion, arterial dissection, aneurysms, and other common vascular diseases. Studies have shown that RNA m6A plays an independent role in the regulation of these high-risk factors. In this review, we discuss the regulatory role of RNA m6A modifications in different high-risk factors. The associations between m6A methylation and risk factors for vascular diseases are presented in Table 2.

**Table 2:** Association between RNA m6A methylation and risk factors of vascular diseases

Risk factor	m6A-related molecules	Expression	m6A levels	Main function	Reference
Obesity	m6A-SNP rs6859 (3ʹUTR-PVRL2)	–	–	Increases LDL, total cholesterol, and triglycerides Causes lipid metabolism dysfunction	[65]
	zfp217	Upregulated	Reduced	Decreases m6A-YTHDF2, leading to obesity	[66]
	FTO	Downregulated	Increased	Decreases total cholesterol and LDL Suppresses the formation of atherosclerotic plaques	[67]
		Upregulated	Reduced	Promotes the formation and differentiation of adipocytes	[68]
	FTO rs9939609 variant	–	–	Increases triglyceride, SCD40L, visfatin, homocysteine, and total cholesterol levels Increases body mass index	[69, 70]
	FTO rs1421085 variant	–	–	Promotes macronutrient intake, obesity, and T2DM	[71]
T2DM	FTO	Upregulated	Reduced	Increases FOX1, G6PC, and DGAT2, leading to dysfunction of lipid and glucose metabolism	[72]
	IGF1-AKT-PDX1	Downregulated	Reduced	Inhibits β-cell arrest and insulin secretion, leading to glucose intolerance and T2DM	[74]
	METTL14	Upregulated	Increased	Affects the differentiation and function of β-cells	[75]
Hypertension	m6A-SNPs rs9847953 and rs197922	–	–	Affects blood pressure regulation	[76]
	MC4R rs17782313 variant	–	–	Affects diastolic pressure and blood pressure	[77]
	FTO	Downregulated	Increased	Protects against tachycardia, hypertension, and vascular resistance	[78]
Atheroslerosis	METTL3	Upregulated	Increased	Increases m6A-FOXO1, increasing ICAM-1 and VCAM-1, leading to atherosclerosis	[79, 79]
	METTL14	Upregulated	Increased	Increased by TNF-α, leads to atherosclerosis	[79]
	FTO rs9939609 variant	–	–	Important risk factor of atherosclerosis	[70]

#### Obesity

Obesity is a pathological change caused by an excessive intake of exogenous lipids or a dysfunction of lipid metabolism. Mo et al*.* [65] used the GWAS database to investigate the effect of m6A-associated single-nucleotide polymorphisms (SNPs) on lipid metabolism. The results have shown that the rs6859 variant at the 3ʹUTR of PVRL2 led to an increase in low-density lipoprotein (LDL), total cholesterol, and triglycerides, and to lipid metabolism dysfunction [65].

Several studies have shown that abnormal variations in FTO expression are closely related to obesity. A recent study carried out by Song et al*.* showed that abnormal increase in zinc-finger protein 217 (zfp217) led to abnormal increase in FTO, which in turn caused a decrease in m6A-YTHDF2, ultimately leading to obesity [66]. In the study by Mo et al*.*, inhibition of the expression of FTO induced a decrease in total cholesterol and LDL, and suppressed the formation of atherosclerotic plaques [67]. Increased FTO expression promotes the formation and differentiation of adipocytes by increasing the expression of Runt-related transcription factor 1 [68]. Studies have also shown that FTO variants affect the morbidity of obesity. In post-menopausal women, FTO rs9939609 variant were prevalent and elicited higher triglyceride, SCD40L, visfatin, homocysteine, and total cholesterol levels as well as higher body mass indices [69, 70]. The FTO rs1421085 (C > T) variant led to higher macronutrient intake, obesity, and T2DM [71]. Due to the role of FTO in obesity, drugs targeting FTO have been developed.

#### Type 2 diabetes mellitus

T2DM often leads to intimal injury, abnormal thickening, and occlusion of peripheral blood vessels. Therefore, it is one of the high-risk factors for ischemic injury and stroke.

In a study by Yang et al*.*, FTO was shown to play an important role in the development of T2DM. In that study, FTO induced an increase in the expression of forkhead box O1 (FOXO1), glucose-6-phosphatase (G6PC), and diacylglycerol O-acyltransferase 2 (DGAT2), which in turn lead to the dysfunction of lipid and glucose metabolism [72]. In FTO knockout mice, a high-fat diet led to glucose intolerance, insulin resistance, and hypertension [73].

Studies have found that RNA m6A modifications are closely related to the function and number of pancreatic β-cells. These, in turn, are key factors in glucose metabolism. β-cell arrest and insulin secretion are inhibited by decreased m6A modifications in insulin-like growth factor 1 (IGF1)-Akt-pancreatic and duodenal homeobox 1 (PDX1) transcripts [74]. METTL14 plays a key role in the development of T2DM by affecting the differentiation and function of β-cells; this, in turn, leads to glucose intolerance and T2DM. Additionally, METTL14 is associated with β-cell death and inflammation [75].

#### Hypertension

In recent years, with the fast economic growth and global improvement of living standards, the morbidity of hypertension as well as its complications, including CHD, AD, and aortic aneurysm, has increased. Previous studies have shown that RNA m6A plays an important role in the development of hypertension.

Mo et al*.* analysed data from GWAS to find a relationship between m6A-associated SNPs and hypertension. A total of 1236 SNPs, such as rs9847953 and rs197922, were found to be associated with blood pressure regulation and most of them were modified by m6A methylation [76]. A recent study by Marcadenti et al*.* found that, in men with hypertension, the m6A-associated SNP rs17782313 in the melanocortin 4 receptor (MC4R) had a negative association with diastolic pressure and mean blood pressure [77].

The eraser FTO also plays a critical role in blood pressure regulation. A decrease in FTO expression leads to protection against tachycardia, hypertension, and vascular resistance. Recent studies have also found that the pathogenesis of hypertension may be related to a change in m6A methylation levels in peripheral cells in microvessels [78].

#### Atherosclerosis

Studies have revealed that atherosclerosis has a close relationship with lipid metabolism and inflammation. In a study by Jian et al., ApoE knockout mice were used to investigate a relationship between RNA m6A modification and atherosclerosis. Their study revealed that FOXO1 m6A methylation was increased by METTL3 and recognized by YTHDF1. This resulted in an increase in intercellular adhesion molecule 1 (ICAM-1) and vascular cell adhesion molecule 1 (VCAM-1), leading to mononuclear/endothelial cell adhesion and atherosclerosis [35, 79]. Tumour necrosis factor-α (TNF-α) is an inflammatory factor that activates and enhances the inflammatory response. Recent studies have shown that TNF-α increases METTL14 expression, which results in endothelial inflammation and atherosclerosis [79]. Additionally, Aijala et al. have found that rs9939609, an abnormal variant of FTO, is an important risk factor of atherosclerosis [70].

### RNA m6A modifications in vascular disease

Studies have shown that the development of many vascular diseases is associated with RNA m6A modifications. We discuss these associations, which are further presented in Table 3.

**Table 3:** Association between m6A methylation and vascular diseases

Vascular diseases	m6A-related molecules	Expression	m6A levels	Main function	Reference
Coronary heart disease	METTL14	Upregulated	Increased	Aggravates vascular calcification	[80]
	FTO	Upregulated	Reduced	Decreases m6A modification of SERCA2a, MY617, and RY2, reducing fibrosis in infarcted areas	[70]
	FTO rs9939609 variant	–	–	Regulates CHD morbidity	[70]
	SNP rs12286 (ADAMTS7)	–	–	Affects RNA m6A modification	[81]
Stroke	FTO	Downregulated	Increased	Modulates poststroke biological processes (inflammation, apoptosis, and transcriptional regulation)	[83]
Aortic aneurysm	FTO	Downregulated	Increased	Induces the formation of aneurysmal smooth muscle cells, macrophage infiltration, and neovascularization	[84]
	METTL14 and YTHDF3	Upregulated	Increased	Induces aneurysmal smooth muscle cell formation, macrophage infiltration, and neovascularization	[84]
Aortic Dissection	METTL3	Upregulated	Increased	Affects hypoxia stress, inducing the differentiation of adipose-derived stem cells into smooth muscle cells Regulates macrophage differentiation and T cells number and function	[86,87
	WTAP	Upregulated	Increased	Participates in a protein complex that affects smooth muscle cell and endothelial cell proliferation and apoptosis	[89]
	HNRNPA2/B1	Upregulated	–	Affects smooth muscle cell differentiation, increasing systolic type smooth muscle cells	[90]
	YTHDF2	Downregulated	–	Decreases inflammation and increases vascular reconstruction and metastatic progression	[91]

#### Coronary heart disease

CHD is caused by coronary luminal narrowing or occlusion due to abnormal lipid metabolism, local vascular wall inflammatory response, and other triggers. Previous studies have shown increased m6A modifications and lowered FTO expression in tissues subjected to myocardial infarction. RNA m6A modifications regulate cardiac contractility, cardiomyocyte differentiation, and metabolism. A study by Chen et al*.* has shown that increased METTL14 expression leads to an increase in m6A, which in turn aggravates vascular calcification [80]. Elevated FTO reduces m6A methylation of SERCA2a, MY617, and RY2, and increases the stability of these mRNAs, improving cardiac contractility and reducing fibrosis in the infarcted area [70].

On the other hand, genetic polymorphisms in FTO have been shown to influence the development of CHD. A study by Aijala et al*.* has shown that the FTO rs9939609 (T > A) variant regulates the morbidity of CHD [70]. The SNP rs12286 at the 3ʹUTR of ADAMTS7 is associated with CHD by affecting the m6A modification of RNA [81].

The detection of m6A modifications on RNA can be used as a potential diagnostic marker for myocardial infarction. A study by Saxena et al*.* has demonstrated that m6A methylation of specific mRNAs is a candidate marker for myocardial infarction diagnosis [82].

#### Stroke

Stroke is caused by intracranial vascular stenosis or occlusion. Previous studies have shown that RNA m6A modification is closely related to stroke. However, research on this topic is still in its infancy. Chokkalla et al*.* have shown that many poststroke biological processes, such as inflammation, apoptosis, and transcriptional regulation, might be modulated by differential expression levels of FTO. RNA m6A modification may be a relevant indicator of poststroke pathophysiology [83].

#### Aortic aneurysm

An aortic aneurysm is an enlargement of the aorta, diagnosed when its diameter exceeds 50% of the normal vascular diameter. Aortic aneurysms can lead to aortic rupture and death. Several underlying causes may exist. Previous studies have shown that RNA m6A modification has a positive relationship with abdominal aortic aneurysm formation and rupture [84]. FTO decrease, YTHDF3 increase, or METTL14 increase may lead to the formation of aneurysmal smooth muscle cells (SMCs), macrophage infiltration, and neovascularization; these pathological changes may elicit the development of an aneurysm [84].

#### Aortic dissection

Aortic dissection is another life-threatening vascular emergency. At present, its exact molecular mechanism is unclear. Studies have shown that the main pathological changes associated with AD are inflammation of vascular tissues, increased apoptosis of ECs and SMCs, decreased cell proliferation, decreased extracellular matrix, and congenital dysplasia of connective tissue [85].

Previous studies have shown that METTL3 plays an important role in the development of AD. METTL3 affects hypoxia stress, which promotes the differentiation of adipose-derived stem cells into SMCs [86]. A recent study by Liu et al*.* has shown that macrophage differentiation and the number and function of T cells can be regulated by METTL3 through modulation of STAT1 expression and the IL-2–STAT5 signaling pathway [87].

In a study performed using human umbilical vein endothelial cells, WTAP combined with Hakai, Virilizer homolog, KIAA0853, RBM15, BCLAF1, and THRAP3 to form a protein complex that functioned as an RNA processing machine and affected cell proliferation [88]. Subsequent research has shown that WTAP exerts different effects on the proliferation and apoptosis of SMCs and ECs. Increased WTAP causes an increase in EC proliferation and a decrease in SMC proliferation; decreased WTAP causes a decrease in EC proliferation, an increase in SMC proliferation, and an increase in DNA synthesis [89].

Readers also play an important role in the formation of AD. In a study by Wang et al*.*, HNRNPA2/B1 affected the differentiation of SMCs, which led to an increase in systolic type SMCs. Decreases in systolic type SMCs are a key factor in AD formation. Therefore, HNRNPA2/B1 maybe a potential target for the treatment of vascular degenerative disease [90]. Additionally, a decrease in YTHDF2 has been shown to lead to reduced inflammation, enhanced vascular reconstruction, and metastatic progression [91].

### Techniques for the detection of RNA m6A modifications

RNA m6A methylation was first identified in 1974; however, it was rediscovered in 2012 with the emergence of next-generation sequencing technology that enabled its detection through the transcriptome [17, 18]. In recent years, with the improvement in the technology for detection of RNA m6A modifications, research on m6A has deepened. Most of the current knowledge from RNA research is stored in the RNA epitranscriptome collection (REPIC) database [92]. In this review, common technologies for the detection of RNA m6A modifications are discussed.

Methylated RNA immunoprecipitation sequencing (MeRIP-seq and m6A-seq) is the oldest and most widely used method to detect m6A-methylated RNA. Random RNA fragmentation, m6A-specific methylated RNA immunoprecipitation, and next-generation sequencing are the basis of the MeRIP-seq and m6A-seq technique. Despite its robust technology, it presents several disadvantages, including: difficulty in accurately locating m6A, antibody bias, difficult data analysis, low reproducibility, and the requirement of large amounts of RNA [93, 94].

Photo-crosslinking-assisted m6A sequencing (PA-m6A-seq), m6A individual-nucleoside-resolution cross-linking and immunoprecipitation (miCLIP), m6A cross-linking immunoprecipitation (m6A-CLIP), and m6A-level and isoform-characterization sequencing (m6A-LAIC-seq) were subsequently established. Their accuracy gradually improved and the required sample amounts gradually decreased; however, because the use of antibodies is still required, antibody bias cannot be avoided [95–99].

Site-specific cleavage and radioactive-labelling followed by ligation-assisted extraction and thin-layer chromatography (SCARLET), RNA-endoribonuclease-facilitated sequencing (m6A-REF-seq), m6A-sensitive RNA digestion and sequencing (MASTER-seq), and deamination adjacent to RNA modification targets (DART-seq) are recent, quantitative, and antibody-free techniques. Their detection ability is accurate and fast, and the amount of RNA required for each detection is low. However, these technologies are still in their primary stages and cannot be used on a large scale [100–103].

## Conclusion and future prospects

Previous studies have shown that RNA m6A modifications play a critical role in the regulation of vascular diseases. However, the research on m6A-methylated RNA is still in its infancy and many unknown fields need to be further explored. First, available studies address individual components of the m6A regulatory system; research on the interaction between different components is lacking. Second, the earlier studies were performed at the cellular and small-animal level; further large animal-level studies and clinical research need to be performed. Third, although several technologies for the detection of m6A-methylated RNA have been recently developed, many are still in their primary stage and cannot be widely used. Fourth, many RNA epigenetic modifications have already been identified. The interrelationship between RNA methylation and other RNA genetic modifications is unclear and considered a future research direction.

In conclusion, RNA m6A modifications, as the most common cellular process of RNA regulation, participate in a variety of biological functions and play an important role in epigenetics. A large amount of evidence shows that RNA N6-methyladenosine modifications play a key role in the morbidity caused due to vascular diseases. Further research on the relationship between RNA N6-methyladenosine modifications and vascular diseases is necessary to understand pathophysiological mechanisms at the gene level and to provide new tools for the diagnosis and treatment of vascular diseases.

## Data Availability

All data generated or analysed during this study are included in this published article.

## Abbreviations

2ʹ-OMe: 2ʹ-O-methylation
AD: Aortic dissection
ALKBH5: α-Ketoglutarate-dependent dioxygenase AlkB homolog 5
CHD: Coronary heart disease
DART-seq: Deamination adjacent to RNA modification targets
DGAT2: Diacylglycerol O-acyltransferase 2
DSP: Desmoplakin
ECs: Endothelial cells
eIF-3: Eukaryotic initiation factor 3
FOXO1: Forkhead box O1
FTO: Fat-mass and obesity-associated protein
G6PC: Glucose-6-phosphatase
HNRNP: Heterogeneous nuclear ribonucleoprotein family
ICAM-1: Intercellular adhesion molecule 1
IGF1: Insulin-like growth factor 1
IGF2BP1: Insulin-like growth factor 2 mRNA binding protein 1
IGF2BP3: Insulin-like growth factor 2 mRNA binding protein 3
LDL: Low-density lipoprotein
m1A: N1-methyladenosine
m5C: 5-Methylcytosine
m6A: N6-methyladenosine
m6A-CLIP: M6A cross-linking immunoprecipitation
m6A-LAIC-seq: M6A-level and isoform-characterization sequencing
m6A-REF-seq: RNA-endoribonuclease-facilitated sequencing
m6Am: N1-methyladenosine
m7G: 7-Methylguanosine
MASTER-seq: M6A-sensitive RNA digestion and sequencing
MC4R: Melanocortin 4 receptor
MeRIP-seq: Methylated RNA immunoprecipitation sequencing
METTL14: Methyltransferase-like 14
METTL16: Methyltransferase-like 16
METTL3: Methyltransferase-like 3
miCLIP: M6A individual-nucleoside-resolution cross-linking and immunoprecipitation
mRNA: Messenger RNA
ncRNA: Non-coding RNA
PA-m6A-seq: Photo-crosslinking-assisted m6A sequencing
PDX1: Pancreatic and duodenal homeobox 1
Prr2a: Proline rich coil 2A protein
RBM15: RNA-binding motif protein 15
RBM15B: RNA-binding motif protein 15B
REPIC: RNA epitranscriptome collection
rRNA: Ribosomal RNA
SCARLET: Site-specific cleavage and radioactive-labelling followed by ligation-assisted extraction and thin-layer chromatography
SMCs: Smooth muscle cells
SNP: Single-nucleotide polymorphism
T2DM: Type 2 diabetes mellitus
TNF-α: Tumor necrosis factor-α
tRNA: Transfer RNA
UTR: Untranslated region
VCAM-1: Vascular cell adhesion molecule 1
VIRMA: Vir-like m6A methyltransferase associated
WTAP: Wilms tumor 1-associated protein
zfp217: Zinc-finger protein 217

## References

[1] Meyer KD, Jaffrey SR (2014). The dynamic epitranscriptome: N6-methyladenosine and gene expression control. Nat Rev Mol Cell Biol.

[2] Wang X, Lu Z, Gomez A, Hon GC, Yue Y, Han D (2014). N6-methyladenosine-dependent regulation of messenger RNA stability. Nature.

[3] Edupuganti RR, Geiger S, Lindeboom RGH, Shi H, Hsu PJ, Lu Z (2017). N6-methyladenosine (m6A) recruits and repels proteins to regulate mRNA homeostasis. Nat Struct Mol Biol.

[4] Boccaletto P, Machnicka MA, Purta E, Piatkowski P, Baginski B, Wirecki TK (2018). MODOMICS: a database of RNA modification pathways 2017 update. Nucleic Acids Res.

[5] Huang H, Weng H, Chen J (2020). The Biogenesis and Precise Control of RNA m6A Methylation. Trends Genet.

[6] Xuan JJ, Sun WJ, Lin PH, Zhou KR, Liu S, Zheng LL, et al. RMBase v2.0: deciphering the map of RNA modifications from epitranscriptome sequencing data. Nucleic Acids Res. 2018;46 (D1), D327–d34. http://doi.org/10.1093/nar/gkx934. 10.1093/nar/gkx934 PMC5753293 29040692

[7] Chen J, Du B (2019). Novel positioning from obesity to cancer: FTO, an m(6)A RNA demethylase, regulates tumour progression. J Cancer Res Clin Oncol.

[8] He L, Li H, Wu A, Peng Y, Shu G, Yin G (2019). Functions of N6-methyladenosine and its role in cancer. Mol Cancer.

[9] Desrosiers R, Friderici K, Rottman F (1974). Identification of methylated nucleosides in messenger RNA from Novikoff hepatoma cells. Proc Natl Acad Sci USA.

[10] Mongelli A, Atlante S, Bachetti T, Martelli F, Farsetti A, Gaetano C. Epigenetic signaling and RNA regulation in cardiovascular diseases. Int J Mol Sci, 2020, 21(2). http://doi.org/10.3390/ijms21020509. 10.3390/ijms21020509 PMC7014325 31941147

[11] Lu D, Thum T (2019). RNA-based diagnostic and therapeutic strategies for cardiovascular disease. Nat Rev Cardiol.

[12] Zhi Y, Xu C, Sui D, Du J, Xu FJ, Li Y (2019). Effective Delivery of Hypertrophic miRNA Inhibitor by Cholesterol-Containing Nanocarriers for Preventing Pressure Overload Induced Cardiac Hypertrophy. Adv Sci (Weinh).

[13] Feng Y, Xu W, Zhang W, Wang W, Liu T, Zhou X (2019). LncRNA DCRF regulates cardiomyocyte autophagy by targeting miR-551b-5p in diabetic cardiomyopathy. Theranostics.

[14] Townsend N, Wilson L, Bhatnagar P, Wickramasinghe K, Rayner M, Nichols M (2016). Cardiovascular disease in Europe: epidemiological update 2016. Eur Heart J.

[15] Arnett DK, Blumenthal RS, Albert MA, Buroker AB, Goldberger ZD, Hahn EJ (2019). 2019 ACC/AHA guideline on the primary prevention of cardiovascular disease: executive summary: a report of the American College of Cardiology/American Heart Association Task Force on clinical practice guidelines. Circulation.

[16] Roth GA, Forouzanfar MH, Moran AE, Barber R, Nguyen G, Feigin VL (2015). Demographic and epidemiologic drivers of global cardiovascular mortality. N Engl J of Med.

[17] Dominissini D, Moshitch-Moshkovitz S, Schwartz S, Salmon-Divon M, Ungar L, Osenberg S (2012). Topology of the human and mouse m6A RNA methylomes revealed by m6A-seq. Nature.

[18] Meyer KD, Saletore Y, Zumbo P, Elemento O, Mason CE, Jaffrey SR (2012). Comprehensive analysis of mRNA methylation reveals enrichment in 3’ UTRs and near stop codons. Cell.

[19] Cao G, Li HB, Yin Z, Flavell RA (2016). Recent advances in dynamic m6A RNA modification. Open Biol.

[20] Meyer KD, Jaffrey SR (2017). Rethinking m6A readers, writers, and erasers. Annu Rev Cell Dev Biol.

[21] Peer E, Rechavi G, Dominissini D (2017). Epitranscriptomics: regulation of mRNA metabolism through modifications. Curr Opin Chem Biol.

[22] Zhao BS, Roundtree IA, He C (2017). Post-transcriptional gene regulation by mRNA modifications. Nat Rev Mol Cell Biol.

[23] Shi H, Wei J, He C (2019). Where, when, and how: contextdependent functions of RNA methylation writers, readers, and erasers. Mol Cell.

[24] Liu J, Harada BT, He C (2019). Regulation of gene expression by N6-methyladenosine in Cancer. Trends Cell Biol.

[25] Patil DP, Chen CK, Pickering BF, Chow A, Jackson C, Guttman M (2016). m6A RNA methylation promotes XIST-mediated transcriptional repression. Nature.

[26] Xiao W, Adhikari S, Dahal U, Chen YS, Hao YJ, Sun BF (2016). Nuclear m6A reader YTHDC1 regulates mRNA splicing. Mol Cell.

[27] Pan Y, Ma P, Liu Y, Li W, Shu Y (2018). Multiple functions of m6A RNA methylation in cancer. J Hematol Oncol.

[28] Meyer KD, Patil DP, Zhou J, Zinoviev A, Skabkin MA, Elemento O (2015). 5′ UTR m6A promotes Cap-independent translation. Cell.

[29] Wang X, Zhao BS, Roundtree IA, Lu Z, Han D, Ma H (2015). N6-Methyladenosine modulates messenger RNA translation efficiency. Cell.

[30] Bokar JA, Shambaugh ME, Polayes D, Matera AG, Rottman FM. Purification and cDNA cloning of the AdoMet-binding subunit of the human mRNA (N6-adenosine)-methyltransferase. RNA. 1997; 3(11): 1233–47. PMCID: PMC1369564. PMC1369564 9409616

[31] Song H, Feng X, Zhang H, Luo Y, Huang J, Lin M (2019). METTL3 and ALKBH5 oppositely regulate m6A modification of TFEB mRNA, which dictates the fate of hypoxia/reoxygenation-treated cardiomyocytes. Autophagy.

[32] Wang P, Doxtader KA, Nam Y (2016). Structural Basis for Cooperative Function of Mettl3 and Mettl14 Methyltransferases. Mol Cell.

[33] Schöller E, Weichmann F, Treiber T, Ringle S, Treiber N, Flatley A (2018). Interactions, localization, and phosphorylation of the m6A generating METTL3-METTL14-WTAP complex. RNA.

[34] Yao MD, Jiang Q, Ma Y, Liu C, Zhu CY, Sun YN (2020). Role of METTL3-dependent N6-methyladenosine mRNA modification in the promotion of angiogenesis. Mol Ther.

[35] Chamorro-Jorganes A, Sweaad WK, Katare R, BesnierM, Anwar M, Beazley-Long N, et al. METTL3 regulates angiogenesis by modulating let-7e-5p and miRNA-18a-5p expression in endothelial cells. Arterioscler Thromb Vasc Biol. 2021;41(6):325–37. http://doi.org/10.1161/ATVBAHA.121.316180. 10.1161/ATVBAHA.121.316180 33910374

[36] Ping XL, Sun BF, Wang L, Xiao W, Yang X, Wang WJ (2014). Mammalian WTAP is a regulatory subunit of the RNA N6-methyladenosine methyltransferase. Cell Res.

[37] Liu J, Yue Y, Han D, Wang X, Fu Y, Zhang L (2014). A METTL3-METTL14 complex mediates mammalian nuclear RNA N6-adenosine methylation. Nat Chem Biol.

[38] Wang LJ, Xue Y, Li H, Huo R, Yan Z, Wang J (2020). Wilms’ tumour 1-associating protein inhibits endothelial cell angiogenesis by m6A-dependent epigenetic silencing of desmoplakin in brain arteriovenous malformation. J Cell Mol Med.

[39] Zhou X, Stuart A, Dettin LE, Rodriguez G, Hoel B, Gallicano GI (2004). Desmoplakin is required for microvascular tube formation in culture. J Cell Sci.

[40] Garrod D, Chidgey M (2008). Desmosome structure, composition and function. Biochim Biophys Acta.

[41] Nielsen CM, Huang L, Murphy PA, Lawton MT, Wang RA (2016). Mouse models of cerebral arteriovenous malformation. Stroke.

[42] Warda AS, Kretschmer J, Hackert P, Lenz C, Urlaub H, Hobartner C, et al. Human METTL16 is a N(6)-methyladenosine (m(6)A) methyltransferase that targets pre-mRNAs and various non-coding RNAs. EMBO Rep. 2017;18 (11): 2004–14. http://doi.org/10.15252/embr.201744940. 10.15252/embr.201744940 PMC5666602 29051200

[43] Ruszkowska A, Ruszkowski M, Dauter Z, Brown JA (2018). Structural insights into the RNA methyltransferase domain of METTL16. Sci Rep.

[44] Mendel M, Chen KM, Homolka D, Gos P, Pandey RR, McCarthy AA, et al. Methylation of structured RNA by the m6A writer METTL16 is essential for mouse embryonic development. Mol Cell. 2018;71 (6): 986–1000, e11. http://doi.org/10.1016/j.molcel.2018.08.004. 10.1016/j.molcel.2018.08.004 PMC6162343 30197299

[45] Yue Y, Liu J, Cui X, Cao J, Luo G, Zhang Z (2018). VIRMA mediates preferential m6A mRNA methylation in 3’UTR and near stop codon and associates with alternative polyadenylation. Cell Discov.

[46] McTaggart JS, Lee S, Iberl M, Church C, Cox RD, Ashcroft FM (2011). FTO is expressed in neurones throughout the brain and its expression is unaltered by fasting. PLoS ONE.

[47] Ho AJ, Stein JL, Hua X, Lee S, Hibar DP, Leow AD (2010). A commonly carried allele of the obesity-related FTO gene is associated with reduced brain volume in the healthy elderly. Proc Natl Acad Sci USA.

[48] Gao X, Shin YH, Li M, Wang F, Tong Q, Zhang P (2010). The fat mass and obesity associated gene FTO functions in the brain to regulate postnatal growth in mice. PLoS ONE.

[49] Gulati P, Avezov E, Ma M, Antrobus R, Lehner P, O’Rahilly S (2014). Biosci Rep.

[50] Mauer J, Luo X, Blanjoie A, Jiao X, Grozhik AV, Patil DP (2017). Reversible methylation of m6Am in the 5’ cap controls mRNA stability. Nature.

[51] Frayling TM, Timpson NJ, Weed MN, Zeggini E, Freathy RM, Lindgren CM (2007). A common variant in the FTO gene is associated with body mass index and predisposes to childhood and adult obesity. Science.

[52] Church C, Moir L, McMurray F, Girard C, Ranks GT, Teboul L (2010). Overexpression of FTO leads to increased food intake and results in obesity. Nat Genet.

[53] Kang H, Zhang Z, Yu L, Li Y, Liang M, Zhou L (2018). FTO reduces mitochondria and promotes hepatic fat accumulation through RNA demethylation. J Cell Biochem.

[54] Ding C, Zou Q, Ding J, Ling M, Wang W, Li H (2018). Increased N6-methyladenosine causes infertility is associated with FTO expression. J Cell Physiol.

[55] Zheng G, Dahl JA, Niu Y, Fedorcsak P, Huang CM, Li CJ (2013). ALKBH5 is amammalian RNA demethylase that impacts RNA metabolism and mouse fertility. Mol Cell.

[56] Xu C, Wang X, Liu K, Roundtree IA, Tempel W, Li YJ (2014). Structural basis for selective binding of m6A. RNA by the YTHDC1 YTH domain. Nat Chem Biol.

[57] Huang X, Zhang H, Guo X, Zhu ZX, Cai HB, Kong XY (2018). Insulin-like growth factor 2 mRNA-binding protein 1 (IGF2BP1) in cancer. J Hematol Oncol.

[58] Huang H, Weng H, Sun W, Qin X, Shi H, Wu H (2018). Recognition of RNA N6-methyladenosine by IGF2BP proteins enhances mRNA stability and translation. Nat Cell Biol.

[59] Wu R, Li A, Sun B, Sun JG, Zhang J, Zhang T (2019). A novel m6A reader Prrc2a controls oligodendroglial specification and myelination. Cell Res.

[60] Li A, Chen YS, Ping XL, Yang X, Xiao W, Yang Y (2017). Cytoplasmic m6A reader YTHDF3 promotes mRNA translation. Cell Res.

[61] Kretschmer J, Rao H, Hackert P, Sloan KE, Hobartner C, Bohnsack MT (2018). The m6A reader protein YTHDC2 interacts with the small ribosomal subunit and the 5’-3’ exoribonuclease XRN1. RNA.

[62] Hsu PJ, Zhu Y, Ma H, Guo Y, Shi X, Liu Y (2017). Ythdc2 is an N6- methyladenosine binding protein that regulates mammalian spermatogenesis. Cell Res.

[63] Wu B, Su S, Patil DP, Liu H, Gan J, Jaffrey SR (2018). Molecular basis for the specific and multivariant recognitions of RNA substrates by human hnRNP A2/ B1. Nat Commun.

[64] Liu N, Zhou KI, Parisien M, Dai Q, Diatchenko L, Pan T (2017). N6-methyladenosine alters RNA structure to regulate binding of a low-complexity protein. Nucleic Acids Res.

[65] Mo X, Lei S, Zhang Y, Zhang H (2019). Genome-wide enrichment of m6A associated single-nucleotide polymorphisms in the lipid loci. Pharmacogenomics J.

[66] Song T, Yang Y, Wei H, Xie X, Lu J, Zeng Q (2019). Zfp217 mediates m6A mRNA methylation to orchestrate transcriptional and post-transcriptional regulation to promote adipogenic differentiation. Nucleic Acids Res.

[67] Mo C, Yang M, Han X, Li J, Gao G, Tai H (2017). Fat mass and obesity-associated protein attenuates lipid accumulation in macrophage foam cells and alleviates atherosclerosis in apolipoprotein E-deficient mice. J Hypertens.

[68] Merkestein M, Laber S, McMurray F, Andrew D, Sachse G, Sanderson J (2015). FTO influences adipogenesis by regulating mitotic clonal expansion. Nat Commun.

[69] Chedraui P, Perez-Lopez FR, Escobar GS, Caicedo JAE, Guevara MM, Genazzani AR (2016). Polymorphisms of the FTO and MTHFR genes and vascular inflammatory and metabolic marker levels in postmenopausal women. J Endocrinol Invest.

[70] Aijala M, Ronkainen J, Huusko T, Malo E, Savolainen ER, Savolainen MJ (2015). The fat mass and obesityassociated (FTO) gene variant rs9939609 predicts long-term incidence of cardiovascular disease and related death independent of the traditional risk factors. Ann Med.

[71] Merino J, Dashti HS, Li SX, Sarnowski C, Justice AE, Graff M (2019). Genome-wide metaanalysis of macronutrient intake of 91,114 European ancestry participants from the cohorts for heart and aging research in genomic epidemiology consortium. Mol Psychiatry.

[72] Yang Y, Shen F, Huang W, Qin S, Huang JT, Sergi C (2019). Glucose Is Involved in the Dynamic Regulation of m6A in Patients with Type 2 Diabetes. J Clin Endocrinol Metab.

[73] Krüger N, Biwer LA, Good ME, Ruddiman CA, Wolpe AG, Delalio LJ (2020). Loss of endothelial FTO antagonizes obesity-induced metabolic and vascular dysfunction. Circ Res.

[74] De Jesus DF, Zhang Z, Kahraman S, Brown NK, Chen M, Hu J (2019). m(6)A mRNA methylation regulates human beta-cell biology in physiological states and in type 2 diabetes. Nat Metab.

[75] Liu J, Luo G, Sun J, Men L, Ye H, He C (2019). METTL14 is essential for betacell survival and insulin secretion. Biochim Biophys Acta Mol basis Dis.

[76] Mo XB, Lei SF, Zhang YH, Zhang H (2019). Examination of the associations between m6A-associated single-nucleotide polymorphisms and blood pressure. Hypertens Res.

[77] Marcadenti A, Fuchs FD, Matte U, Sperb F, Moreira LB, Fuchs SC (2013). Effects of FTO RS9939906 and MC4R RS17782313 on obesity, type 2 diabetes mellitus and blood pressure in patients with hypertension. Cardiovasc Diabetol.

[78] Wu Q, Yuan X, Han R, Zhang H, Xiu R (2019). Epitranscriptomic mechanisms of N6-methyladenosine methylation regulating mammalian hypertension development by determined spontaneously hypertensive tars pericytes. Epigenomics.

[79] Jian D, Wang Y, Jian L, Tang H, Rao L, Chen K (2020). METTL14 aggravates endothelial inflammation and atherosclerosis by increasing FOXO1 N6-methyladeosine modifications. Theranostics.

[80] Chen J, Ning Y, Zhang H, Song N, Gu Y, Shi Y (2019). METTL14-dependent m6A regulates vascular calcification induced by indoxyl sulfate. Life Sci.

[81] Mo XB, Lei SF, Zhang YH, Zhang H (2018). Detection of m(6)A-associated SNPs as potential functional variants for coronary artery disease. EPIGENOMICS-UK.

[82] Saxena R, Weintraub NL, Tang Y (2020). Optimizing cardiac ischemic preconditioning and postconditioning via epitranscriptional regulation. Med Hypotheses.

[83] Chokkalla AK, Mehta SL, Kim T, Chelluboina B, Kim J, Vemuganti R (2019). Transient focal ischemia significantly alters the m6A epitranscriptomic tagging of RNAs in the brain. Stroke.

[84] He Y, Xing J, Wang S, Xin S, Han Y, Zhang J (2019). Increased m6A methylation level is associated with the progression of human abdominal aortic aneurysm. Ann Transl Med.

[85] Macgillivray TE, Gleason TG, Patel HJ, Aldea GS, Bavaria JE, Beaver TM (2022). The society of thoracic surgeons/american association for thoracic surgery clinical practice guidelines on the management of type b aortic dissection. Ann Thorac Surg.

[86] Lin J, Zhu Q, Huang J, Cai R, Kuang Y (2020). Hypoxia promotes vascular smooth muscle cell (VSMC) differentiation of adipose-derived stem cell (ADSC) by regulating Mettl3 and paracrine factors. Stem Cells Int..

[87] Liu Y, Liu Z, Tang H, Shen Y, Gong Z, Xie N (2019). The N6-methyladenosine (m6A)—forming enzyme METTL3 facilitates M1 macrophage polarization through the methylation of STAT1 mRNA. Am J Physiol Cell Physiol.

[88] Horiuchi K, Kawamura T, Iwanari H, Ohashi R, Naito M, Kodama T (2013). Identification of Wilms' tumor 1-associating protein complex and its role in alternative splicing and the cell cycle. J Biol Chem.

[89] Small TW, Pickering JG (2009). Nuclear degradation of Wilms tumor 1-associating protein and survivin splice variant switching underlie IGF-1-mediated survival. J Biol Chem.

[90] Wang G, Xiao Q, Luo Z, Ye S, Xu Q (2012). Functional impact of heterogeneous nuclear ribonucleoprotein A2/B1 in smooth muscle differentiation from stem cells and embryonic arteriogenesis. J Biol Chem.

[91] Hou J, Zhang H, Liu J, Zhao Z, Wang J, Lu Z (2019). YTHDF2 reduction fuels inflammation and vascular abnormalization in hepatocellular carcinoma. Mol Cancer.

[92] Liu S, Zhu A, He C, Chen M (2020). REPIC: a database for exploring the N6- methyladenosine methylome. Genome Biol.

[93] McIntyre ABR, Gokhale NS, Cerchietti L, Jaffrey SR, Horner SM, Mason CE (2020). Limits in the detection of m6A changes using MeRIP/m6A-seq. Sci Rep.

[94] Zeng Y, Wang S, Gao S, Soares F, Ahmed M, Guo H (2018). Refined RIP-seq protocol for epitranscriptome analysis with low input materials. PLoS Biol.

[95] Chen K, Lu Z, Wang X, Fu Y, Luo GZ, Liu N (2015). High-resolution N6 -methyladenosine m6A map using photo-crosslinking-assisted m6A sequencing. Angew Chem Int Ed Eng.

[96] Linder B, Grozhik AV, Olarerin-George AO, Meydan C, Mason CE, Jaffrey SR (2015). Single-nucleotide-resolution mapping of m6A and m6Am throughout the transcriptome. Nat Methods.

[97] Grozhik AV, Linder B, Olarerin-George AO, Jaffrey SR (2017). Mapping m6A at individual-nucleotide resolution using crosslinking and immunoprecipitation (miCLIP). Methods Mol Biol.

[98] Ke S, Alemu EA, Mertens C, Gantman EC, Fak JJ, Mele A (2015). A majority of m6A residues are in the last exons allowing the potential for 3’ UTR regulation. Genes Dev.

[99] Molinie B, Wang J, Lim KS, Hillebrand R, Lu ZX, Van Wittenberghe N (2016). m6A-LAIC-seq reveals the census and complexity of the m6A epitranscriptome. Nat Methods.

[100] Liu N, Parisien M, Dai Q, Zheng G, He C, Pan T (2013). Probing N6 methyladenosine RNA modification status at single nucleotide resolution in mRNA and long noncoding RNA. RNA.

[101] Zhang Z, Chen LQ, Zhao YL, Yang CG, Roundtree IA, Zhang Z (2019). Single-base mapping of m6A by an antibodyindependent method. Sci Adv.

[102] Garcia-Campos MA, Edelheit S, Toth U, Safra M, Shachar R, Viukov S (2019). Deciphering the m6A Code via antibody-independent quantitative profiling. Cell.

[103] Meyer KD (2019). DART-seq: an antibody-free method for global m6A detection. Nat Methods.

